# Targeting the rheumatoid arthritis synovial fibroblast via cyclin dependent kinase inhibition: An early phase trial

**DOI:** 10.1097/MD.0000000000020458

**Published:** 2020-06-26

**Authors:** Stefan Siebert, Arthur G. Pratt, Deborah D. Stocken, Miranda Morton, Amy Cranston, Michael Cole, Sheelagh Frame, Christopher D. Buckley, Wan-Fai Ng, Andrew Filer, Iain B. McInnes, John D. Isaacs

**Affiliations:** a Institute of Infection, Immunity and Inflammation, University of Glasgow, Glasgow; b Translational and Experimental Medicine Institute, Newcastle University and Musculoskeletal Unit, Newcastle upon Tyne Hospitals NHS Foundation Trust, Newcastle upon Tyne; c Leeds Institute of Clinical Trials Research, University of Leeds, Leeds; d Institute of Health and Society, Newcastle University, Newcastle upon Tyne; e Cyclacel Ltd., Dundee; f NIHR Birmingham Biomedical Research Centre, University Hospitals Birmingham NHS Foundation Trust and Institute for Inflammation and Ageing, University of Birmingham, Birmingham; g Kennedy Institute of Rheumatology, Roosevelt Drive, Headington University of Oxford, Oxford, UK.

**Keywords:** Bayesian Continual Reassessment Method, cyclin-dependent kinase, dose-finding, fibroblast, Fleming A’Hern design, rheumatoid arthritis, seliciclib

## Abstract

**Introduction:**

Targeted biologic therapies demonstrate similar efficacies in rheumatoid arthritis despite distinct mechanisms of action. They also exhibit a ceiling effect, with 10% to 20% of patients achieving remission in clinical trials. None of these therapies target synovial fibroblasts, which drive and maintain synovitis. Seliciclib (R-roscovitine) is an orally available cyclin-dependent kinase inhibitor that suppresses fibroblast proliferation, and is efficacious in preclinical arthritis models. We aim to determine the toxicity and preliminary efficacy of seliciclib in combination with biologic therapies, to inform its potential as an adjunctive therapy in rheumatoid arthritis.

**Methods and analysis:**

TRAFIC is a non-commercial, multi-center, rolling phase Ib/IIa trial investigating the safety, tolerability, and efficacy of seliciclib in patients with moderate to severe rheumatoid arthritis receiving biologic therapies. All participants receive seliciclib with no control arm. The primary objective of part 1 (phase Ib) is to determine the maximum tolerated dose and safety of seliciclib over 4 weeks of dosing. Part 1 uses a restricted 1-stage Bayesian continual reassessment method based on a target dose-limiting toxicity probability of 35%. Part 2 (phase IIa) assesses the potential efficacy of seliciclib, and is designed as a single arm, single stage early phase trial based on a Fleming-A’Hern design using the maximum tolerated dose recommended from part 1. The primary response outcome after 12 weeks of therapy is a composite of clinical, histological and magnetic resonance imaging scores. Secondary outcomes include adverse events, pharmacodynamic and pharmacokinetic parameters, autoantibodies, and fatigue.

**Ethics and dissemination:**

The study has been reviewed and approved by the North East - Tyne & Wear South Research Ethics Committee (reference 14/NE/1075) and the Medicines and Healthcare Products Regulatory Agency (MHRA), United Kingdom. Results will be disseminated through publication in relevant peer-reviewed journals and presentation at national and international conferences.

**Trials Registration:** ISRCTN, ISRCTN36667085. Registered on September 26, 2014; http://www.isrctn.com/ISRCTN36667085

**Current protocol version**: Protocol version 11.0 (March 21, 2019)

## 1 Introduction

Rheumatoid arthritis (RA) is a chronic inflammatory arthritis characterized by joint pain, swelling, and damage.^[1]^ RA is a major global public health challenge, with an estimated global prevalence of almost 20 million and significant impact on disability adjusted life years.^[2]^ Around a third of people with RA have stopped working within 2 years of onset and around a half by 10 years, with significant costs to the economy in sick leave and work-related disability.^[3]^ Suboptimally controlled joint inflammation in RA leads to damage, deformity, disability, and impaired quality of life. Chronic inflammation reduces life expectancy by increasing the risk of cardiovascular disease. Advances in RA management strategies and targeted biologic therapies have contributed to significantly improved prognosis and outcomes for people with RA. However, there remains significant unmet clinical need. There is no cure for RA and, even with the best available therapies, only 20% to 30% of patients achieve remission, while 5% to 10% are refractory to all current treatments.^[4]^

Despite modulating different implicated immune pathways, current targeted therapies exhibit remarkably similar response rates in clinical trials. On average, 50% to 60% of participants improve by 20% (American College of Rheumatology (ACR) 20 response), 30% to 40% improve by 50% (ACR50) and 10% to 20% improve by 70% (ACR70). As current therapies target primary immune cells and cytokines, an untested hypothesis is that RA synovial fibroblasts may themselves drive and maintain synovitis, explaining the apparent “ceiling effect” of current therapies, which do not target these cells. RA synovial fluid also contains abundant neutrophils,^[5]^ which secrete pro-inflammatory mediators and tissue-destructive enzymes not specifically targeted by current therapies. A therapy that addresses the abnormal behaviour of RA synovial fibroblasts, particularly with potential additional actions on monocytes/macrophages and neutrophils,^[6,7]^ could have a unique niche in RA management.

Cell proliferation is dependent on orderly progression through the cell cycle, which is regulated by tightly controlled formation and activation of complexes comprising cyclin proteins and cyclin-dependent kinases (CDKs). Dysregulation of cyclin-CDK pathways has been demonstrated in many tumors, and their manipulation consequently exploited to develop anti-cancer drugs.^[8]^ However altered cytokine and tissue destructive enzymes due to dysregulation of these pathways has also been reported in RA synovial fibroblasts.^[9–11]^ Seliciclib (R-roscovitine) is an orally available cyclin dependent kinase inhibitor that selectively targets CDK2, CDK7, and CDK9.^[12,13]^ In addition to directly suppressing fibroblast proliferation by inhibiting CDK2, seliciclib induces p21,^[14,9]^ an endogenous CDK inhibitor whose activity is down-regulated in RA synovial fibroblasts.^[10,15]^ Furthermore, inhibition of CDK7 and CDK9 reduces transcription of MCL1, which reduces viability of synovial fibroblasts, neutrophils, and macrophages.^[16]^ In vitro seliciclib causes growth inhibition and apoptosis of abnormally dividing cells, leaving normally dividing cells unaffected.^[17–20]^ Seliciclib and related CDKIs have demonstrated good efficacy and potency in preclinical models of arthritis.^[21–25]^ Additionally, genetic studies have implicated CDK inhibition as a plausible therapeutic strategy in RA, and support investigation of CDK inhibition in RA.^[26]^ Specific interactions of conventional synthetic disease-modifying anti-rheumatic drugs (csDMARDs) or biologic disease-modifying anti-rheumatic drugs (bDMARDs) with seliciclib are not predicted. Seliciclib has a reported toxicity profile similar to existing csDMARDs used to treat RA, and without the myelosuppression seen with other CDK inhibitors.^[27]^ Preclinical studies suggest combining CDK inhibition with cytokine blockers has additive effects and may not increase immune suppression.^[28]^ Determination of the toxicity profile and preliminary efficacy of seliciclib in combination with bDMARD therapies within a true-to-life pharmacological context (in which concomitant csDMARD use is permitted), will provide important insight into its potential acceptability as an adjunctive therapy in RA.

## 2 Methods and analysis

### 2.1 Overall study design and objectives

TRAFIC is an investigator-led, multi-center, rolling phase Ib/IIa trial investigating the safety, tolerability, and efficacy of seliciclib as an addition to existing bDMARD therapy in patients with moderate-severe RA. All eligible participants will receive seliciclib, the investigational medicinal product (IMP), with no randomization or control arm.

The primary objective of part 1 of the study (phase Ib) is to determine the maximum tolerated dose (MTD) and safety of seliciclib over 4 weeks when given in RA as an addition to existing bDMARD therapy. The MTD was established using a restricted 1-stage Bayesian Continual Reassessment Method (CRM)^[29]^ based on a target dose-limiting toxicity (DLT) probability of 35% (similar to what is observed with methotrexate, the “anchor” drug in RA clinical practice^[30]^), or determination of unacceptable toxicity leading to cessation of the trial. It is planned that up to 7 cohorts of 3 participants each will be treated, with each cohort receiving 1 of 5 possible doses of the IMP. The trial design allows for early stopping if sufficient evidence of MTD has been achieved or if the lowest IMP dose is too toxic. Part 1 of the study has been completed and clinical data will be published in a separate manuscript.

The primary objective of part 2 (phase IIa) is to assess the potential efficacy, using a composite response, of seliciclib as an addition to existing therapy following 12 weeks of treatment. Part 2 is designed as a single arm, single stage early phase trial based on a Fleming–A’Hern design^[31]^ recruiting a total of 18 participants to receive the MTD recommended from part 1 for a maximum of 12 weekly cycles. At the conclusion of part 2, further investigation is warranted if the observed number of clinical responses at least equals a pre-specified critical number according to the statistical design. As this is the first trial investigating seliciclib in this indication as a repurposed drug and given the novel mechanism of action, the decision to continue will also be based on pharmacodynamic (PD) biomarkers and pharmacokinetic (PK) parameters, to avoid rejecting a potentially active drug which has not achieved pragmatic clinical measures of efficacy. A secondary aim, given seliciclib's potentially unique mode of action in RA, is to identify sensitive and robust outcome measures for future RA trials of seliciclib and similar agents in this indication.

When we wrote the initial protocol, there was a significant unmet need for patients who had received tumor necrosis factor (TNF) blockade but who had not reached remission. Whilst the same was true for patients receiving other bDMARDs, more patients were receiving anti-TNF therapy at that stage because this treatment had been licensed for significantly longer than other biologic agents. The initial study protocol was therefore limited to patients receiving TNF inhibitors. However, recently more patients are being “cycled” from a TNF inhibitor to another bDMARD as part of their standard care and existing treatment guidelines, thereby reducing the pool of patients suitable for TRAFIC and significantly impacting on study recruitment. In other words, the “unmet need” has now been shifted downstream to include patients receiving other biologic therapies. We therefore amended the protocol (approved June 2019) to broaden the inclusion criteria to incorporate patients on 2 other bDMARDs (tocilizumab and abatacept) supported by preclinical data.^[28]^ In addition, the 28 joint disease activity score (DAS28) cut-off for inclusion in part 2 of the study was reduced from ≥4.0 to ≥3.2 as this was identified as a barrier to recruitment and 3.2 was felt to be a more clinically relevant cut-off.

### 2.2 Study population

Adults (≥18 years old) fulfilling the 1987 ACR or 2010 ACR/European League Against Rheumatism (EULAR) criteria for RA who are currently taking a bDMARD therapy for at least 3 months as part of standard clinical care and who have ongoing moderate-severe disease activity are eligible. In part 1 of the study, the bDMARD was limited to anti-TNF therapy, which was amended in June 2019 to include abatacept or tocilizumab. The DAS28 cut-off in part 1 was ≥3.2. In part 2, the initial proposed DAS28 cut-off was ≥4.0 plus clinical synovitis in at least 3 joints, which was amended to ≥3.2 plus at least 1 joint amenable to ultrasound-guided synovial biopsy in June 2019. The current protocol allows anti-TNF or 2 other bDMARD therapies (abatacept or tocilizumab) to be used either as monotherapy or with permitted background csDMARDs (methotrexate, sulphasalazine, and hydroxychloroquine).

Full inclusion and exclusion criteria for current protocol v11.0 (March 21, 2019) are shown in Table 1. Potential participants are recruited from rheumatology outpatient departments in the United Kingdom.

**Table 1:**
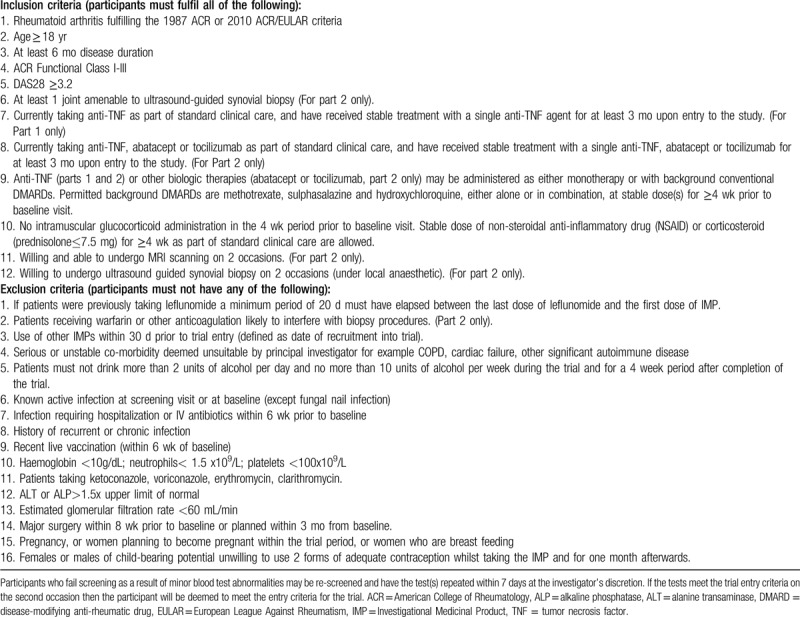
Inclusion and exclusion criteria for current protocol v11.0 (March 21, 2019).

### 2.3 Ethical considerations

The study has been reviewed and given favourable ethical opinion by the North East – Tyne & Wear South Research Ethics Committee (reference number 14/NE/1075). Clinical trial authorization was given by the Medicines and Healthcare Products Regulatory Agency, United Kingdom. The study is being conducted in compliance with Good Clinical Practice, the Research Governance Framework for Health and Social Care, and national legislation implementing the EU Clinical Trials Directive (2001/20/EC) and subsequent amendments. Patients wishing to take part provide written informed consent by signing and dating a trial specific consent form, obtained prior to any study-specific procedures. The study is registered in the ISRCTN registry (identifier 36667085).

### 2.4 Data collection

Data will be collected using standardized data entry and management system. An overview of the schedule of enrolment and timing and types of data collected in parts 1 and 2 of the study are shown in Tables 2 and 3, respectively.

**Table 2:**
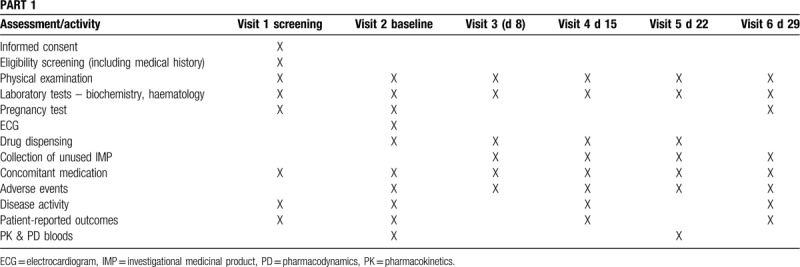
Schedule for enrolment and data collection in part 1.

**Table 3:**
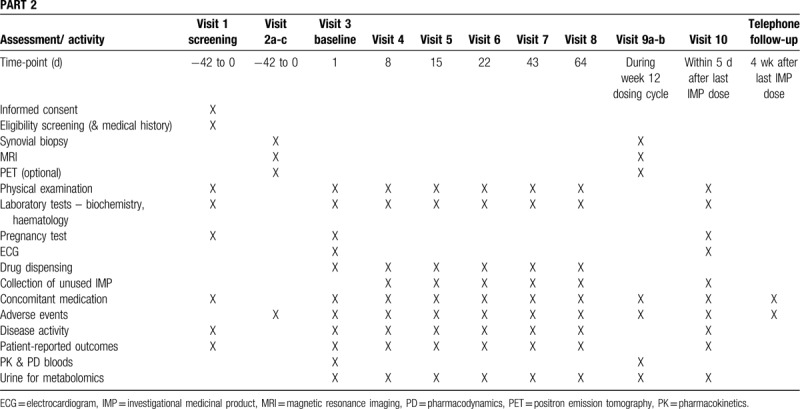
Schedule for enrolment and data collection in part 2.

### 2.5 Outcome measures

#### 2.5.1 Primary outcome measures

Part 1: The MTD is defined as the dose that is closest to the dose at which 35% of participants experience a DLT over a 4-week treatment period with seliciclib. A DLT is defined as the cessation of IMP due to adverse events or reactions (AE/AR) occurring during the treatment period. AEs and ARs are attributed (as definitely, probably, possibly, unlikely, or unrelated) to seliciclib and other therapies and categorised as mild, moderate or severe. These can be either symptomatic AEs or ARs (e.g., nausea) or abnormal laboratory parameters or investigations. DLT is based upon the patient's request to stop treatment, regardless of the severity classification, or an abnormal laboratory parameter necessitating cessation of treatment. In the event of several AEs/ARs contributing to the decision to discontinue IMP, only a single DLT will be recorded for the purposes of dose pathway decision making.

Part 2: Response rate at 12 weeks as a composite measure using clinical scores (EULAR moderate response, ACR20 response),^[32,33]^ histological score (macrophage number in the sublining layer of the synovium)^[34–37]^ and magnetic resonance imaging (MRI) score (rheumatoid arthritis MRI scoring system (RAMRIS)).^[38–40]^ A patient receiving seliciclib is defined as responding if they achieve 2 out of 3 of:

1. EULAR moderate response or ACR20 response
2. A reduction of sublining macrophage number of ≥20% or an absolute mean reduction of >200 macrophages/mm^2^ at the end of dosing
3. A reduction of RAMRIS synovitis of ≥0.5 units or osteitis score of ≥0.2 units at the end of dosing

### 2.6 Secondary outcome measures

Both study parts record and report:

1. all AEs,
2. PD biomarkers in peripheral blood,
3. PK parameters in peripheral blood,
4. relationship between PD and PK markers with each other and with AEs,
5. longitudinal changes in FACIT Fatigue Scale and
6. longitudinal changes in circulating autoantibodies.

Part 2 also records and reports:

1. response rate after 1, 2, 3, 6, and 9 weeks of therapy;
2. relationship between PD and PK markers and efficacy,
3. change in synovial metabolism assessed by fluorodeoxyglucose uptake by inflamed synovium measured by fluorodeoxyglucose positron emission tomography at baseline and at week 12 (optional);
4. change from baseline in urinary metabolomic profiles during treatment; and
5. molecular changes in the synovium consistent with seliciclib PD activity.

### 2.7 Investigational medicinal product

Seliciclib is the IMP. The selected dose range and schedule is based on healthy control and oncology studies in which more than 450 participants have received between 50 and 3600 mg seliciclib per day in a variety of intermittent dosing schedules. Using the selected 4 days per week schedule, the longest treatment period was 111 weeks. The terminal half-life is 3 to 4 hours and primary route of metabolism is via cytochromes 3A4 and 2B6, the major metabolite being much less active. Tolerability in previous studies was related to daily dose and number of consecutive dosing days. DLTs were reversible below 1600 mg of seliciclib daily and included fatigue, nausea and vomiting, elevated liver enzymes, and hypokalaemia. Non-limiting toxicities included anorexia and elevated serum creatinine. Most AEs were mild to moderate in severity, dose-related, and generally occurred during the first 3 weeks of therapy. Such symptoms are familiar to RA patients, either as a manifestation of the disease itself or as side effects of commonly prescribed csDMARDs. Hence the experience to date, particularly lack of myelosuppression, suggests that seliciclib should be acceptable and well tolerated in RA.

The IMP is supplied as a 200 mg strength capsule (white opaque, size 1). Cyclacel Pharmaceutical provides the bulk IMP. Penn Pharmaceuticals Ltd is responsible for treatment packaging and labelling. The IMP is prescribed by a trial clinician according to the protocol and dispensed according to local pharmacy policy. Participants return all IMP supplies in their original packaging (even if empty) to the trial clinician or pharmacist at each visit. All returned or unused IMP is stored in pharmacy until the end of the trial, or until the trial manager has completed appropriate reconciliation.

In part 1, participants receive 200, 400, 600, 800, or 1000 mg seliciclib daily for 4 consecutive days every week for 4 weeks. Participants are assigned to the dose levels in cohorts of 3, with 400 mg seliciclib assigned to the first cohort. Participants in part 1 receive IMP treatment for 4 weekly cycles (in the absence of DLT) and, once the third subject of a cohort has attended their final visit, the chief investigator, principal investigators from each site, trial manager and trial statistician discuss any AEs/ARs and DLTs and make the decision whether or not to open the subsequent cohort, according to the CRM algorithm, with sponsor and Data Monitoring Committee (DMC) approval. An independent DMC undertakes independent review with the purpose of monitoring safety and efficacy endpoints. Dose cannot be escalated or de-escalated by more than 200 mg per cohort, and cohorts are enrolled until the MTD was determined, the maximum sample size reached, or if the lowest dose (200 mg) is determined to be too toxic.

In part 1 of the trial, a DLT triggers patient withdrawal and informs the CRM algorithm. In part 2, seliciclib is withheld in the presence of severe DLTs, until the toxicity resolves to mild, with the exception of anaemia, hypokalaemia, or other laboratory abnormality that can be managed adequately by supplementation or medications. Dosing is recommenced at the next lower dose, and all dose reductions are permanent. In the event of a non-severe DLT, dosing with seliciclib may be withheld until the toxicity resolves. At this point drug is restarted at the same dose unless the treatment delay is 2 weeks or more, in which case a dose reduction is mandated as above.

Participants may not receive any other investigational drugs while participating in this trial but may receive existing standard therapies and supportive treatment. As nausea and vomiting are commonly reported by participants treated with seliciclib, prophylactic use of an anti-emetic is permissible but not mandatory.

### 2.8 Statistical considerations

All analyses are predefined and documented in the statistical analysis plan approved by the chief investigator. The statistical design will be reported in detail in a separate manuscript. Briefly, part 1 will report the recommended MTD, the Bayesian posterior probability of DLT at each dose level (with 90% probability interval) and the posterior probability that the DLT rate at dose level 1 (200 mg) is greater than the target level of 35%. The number of participants experiencing DLT at each dose level, together with the proportion of participants with DLT at that dose level, will be reported. Secondary outcome measures including the relationship of PD biomarkers and PK parameters will be presented graphically.

Part 2 will assess potential efficacy of treatment based on a composite outcome of response following 12 cycles of treatment. Individual components of the composite response outcome and all AEs will also be reported descriptively. Secondary outcome measures including PK parameters and PD biomarkers will be presented graphically.

### 2.9 Sample size

Part 1 includes a maximum of 21 participants based on a maximum of 7 cohorts each consisting of 3 participants. The decision to recruit 3 participants per cohort was based on the proposed 35% DLT rate. The performance of the Bayesian design based on 21 participants was assessed via simulations under several clinically relevant scenarios in terms of accuracy of selecting the true MTD, optimal allocation, and average percentage of participants being treated at a dose higher than the MTD.

Part 2 is based on a Fleming–A’Hern design^[32]^ and assumes a composite response rate to reject seliciclib (p0) <25% and a response rate to investigate seliciclib further (p1) >50%. The justification to investigate seliciclib further is based on observing a minimum critical number of responses. As an early phase trial, the error levels have been inflated but restricted to an acceptable level of 15% alpha (type 1) and 20% beta (type 2). With these stated parameters the target recruitment for part 2a is calculated as 18 participants.

As this is the first trial investigating seliciclib as a repurposed drug for RA and, given the novel mechanism of action, the decision to continue will also be based on PD biomarkers and PK parameters to minimise the risk of rejecting a potentially active drug which has not achieved pragmatic clinical measures of efficacy. In terms of synovial PD biomarkers, the decision to continue will be informed by the following observations and evidence:

1. Polymerase chain reaction of synovial tissue for genes of interest relevant to fibroblast biology, inflammation, cell cycle, and apoptosis, applied to mRNA extracted from synovial tissue;
2. PD effects of seliciclib on the synovial fibroblast using immunohistochemistry and appropriate dual staining with fibroblast markers, including reagents that recognise phospho-specific isoforms of RNA polymerase II and the retinoblastoma protein, which are targets of CDK7/9 and CDK2, respectively.
3. Markers of cell proliferation, such as Ki-67, within the synovial fibroblast population.

If any of these criteria suggest a positive PD effect of seliciclib in the synovium, particularly if the IMP is well tolerated, then a decision may be taken to progress to a further study with a more focussed dose range and using outcome measures informed by the current study.

### 2.10 Trial monitoring

The trial is monitored by an independent DMC consisting of 2 physicians not connected to the trial, 1 acting as chair, and 1 statistician. The purpose of this committee is to undertake independent review and monitor efficacy and safety endpoints. The committee convened prior to the start of recruitment to discuss the dose transition pathway as recommended by the proposed CRM. The DMC members are updated after completion of each cohort in part 1. The DMC also convenes at the end of part 1, during recruitment of part 2 and at trial completion, with additional interim meetings at the request of the DMC members in light of emerging safety data.

A Trial Steering Committee (TSC) provides overall supervision of the trial. The TSC consists of an independent chairperson, an independent clinician plus principal investigators, a Cyclacel representative and lay representation. In addition, representatives from the Trial Management Group, sponsor and funder will be invited. Requests for data sharing will be reviewed and considered by the TSC in conjunction with the chief investigator and sponsor.

The trial may be subject to inspection and audit by The Newcastle upon Tyne Hospitals NHS Foundation Trust under their remit as Sponsor, and other regulatory bodies to ensure adherence to GCP.

### 2.11 Patient and public involvement

Two patient research partners have been involved throughout this project. They have contributed to the initial project design and protocol, reviewed and improved the information sheet for participants, and have taken part in regular project steering group meetings. The patient research partners will review the results of this study, contribute to the drafting of lay summaries for all manuscripts and will advise and participate in the dissemination of results to lay audiences.

### 2.12 Study discontinuation

The trial may be prematurely discontinued on the basis of new safety information, or for other reasons given by the DMC and/or TSC, sponsor, regulatory authority, or Ethics Committee concerned. There are 2 early stopping rules included in part 1 to allow for early termination if:

1. there is a high probability that the posterior probability of DLT at the lowest dose is greater than the target DLT rate, indicating that the lowest dose is too toxic or
2. sufficient patients have already been allocated at the current MTD, which would also be the recommended dose level for the next cohort if the trial continues.

## 3 Discussion

The TRAFIC study aims to repurpose seliciclib to address an area of unmet need in RA. This is the first study of a CDK inhibitor or other therapeutic agent targeting the synovial fibroblast in RA, and employs efficient trial designs that additionally avoid the requirement for control arms in part 1 or part 2 of the study.

While seliciclib has been tested in patients with malignancy,^[27,41]^ the MTD for RA cannot be assumed to be the same. Patients with RA will have received a number of immunomodulating DMARD therapies, anti-inflammatories and analgesics as part of their previous treatment. Furthermore, patients with RA may exhibit a different tolerance to adverse effects than patients with cancer. The MTD dose for seliciclib in patients with RA therefore has to be established in part 1 of this study before assessment of efficacy can be undertaken.

The potential target RA patient population for seliciclib was extensively discussed. There are multiple csDMARD and bDMARD therapies, with established efficacy and safety, available for the treatment of early and moderate-severe active RA. In general, patients who fail to respond to initial csDMARDs are rapidly escalated to biologic therapies. Despite the distinct mechanisms of action of bDMARDs, their efficacy is surprisingly similar in RA clinical trials, including a therapeutic “ceiling” such that only 10% to 20% of patients achieve remission. The hypothesis under test in TRAFIC is that these unexpected phenomena could be explained if a proportion of RA disease activity were attributable to the synovial fibroblast. This cell is an integral element of RA “pannus”, the tissue that erodes bone and cartilage; it also secretes pro-inflammatory cytokines and chemokines, thereby potentially sustaining inflammation in the presence of potent biologic therapies. Current RA therapies do not target synovial fibroblasts, hence the rationale for adding seliciclib to existing therapeutic regimes in patients with a partial response to biologic therapies. The DAS28 cut-offs for inclusion in this study was chosen to reflect ongoing disease activity and for being feasible for recruitment. Part 1 was designed to determine the MTD of seliciclib, and a DAS28 score of ≥3.2 was chosen. In part 2, a higher DAS28 score of ≥4.0 was initially selected to maximise efficacy assessment but subsequently this was reduced to ≥3.2 to improve recruitment. DAS28 scores of ≥3.2 indicate moderate disease activity, with DAS28 <3.2 representing low disease activity^[42]^ and DAS28 <2.6 representing remission.^[43]^

Part 1 utilised a Bayesian model-based CRM dose finding design used more frequently in oncology. CRM designs have been shown to be more efficient than the often used rule-based designs, such as the 3 + 3; CRM designs require fewer patients on average and treat more patients at potentially therapeutic doses at or near to the MTD.^[44]^ In contrast to rule-based designs, CRM designs utilise all available accumulating data to inform decisions regarding dosing for successive patients. Part 2 is designed as a proof of concept trial with a go/no-go decision for a further randomized phase II/III trial. This single arm, single stage, early-phase trial uses the Fleming–A’Hern design which incorporates specification of a desired level of response and a level of response below which the treatment would be considered ineffective.

The part 2 outcomes were chosen to reflect both symptoms and biomarkers. The latter are particularly important for a study design that does not include a placebo or control group. ACR responses are composite scores reflecting swollen and tender joints, an acute phase biomarker, physician, and patient global assessments, an assessment of function (Health Assessment Questionnaire) and an assessment of pain. ACR20 was initially developed to reflect an improvement in phase II/III clinical trials that was unlikely to represent a placebo response. Similarly, a moderate EULAR response is likely to reflect a true benefit. Nonetheless, in a single arm study where all patients receive active treatment, a strong placebo response is anticipated. Hence the inclusion of validated biomarkers based on a reduction of macrophages in the synovial sublining and MRI evidence of improvement in synovitis and/or osteitis. In previous studies, prednisolone and a CCR1 antagonist resulted in 40% to 50% reduction in sublining macrophages over 12 to 14 weeks in controlled studies, while in contrast patients receiving placebo or stable background therapy demonstrated a 10% to 25% increase in sublining macrophages.^[34,36]^ The RAMRIS criteria for MRI improvement reflect those observed in a 12 week phase II study of fostamatinib (Syk kinase inhibitor) in RA, albeit a much larger study than the current one.^[40]^ The RAMRIS score in the placebo group of that study increased (indicating disease progression). We believe that fluorodeoxyglucose positron emission tomography, reflecting synovial metabolism, could also provide a useful outcome measure predicted to reduce after seliciclib. However, as a non-validated outcome measure this is optional for part 2 participants and not included in the Fleming–A’Hern decision process.

TRAFIC is the first trial to use a cell cycle inhibitor to target the synovial fibroblast in patients with RA. It is also innovative in employing CRM to identify the MTD in part 1, and a single arm Fleming–A’Hern design to seek evidence of efficacy in part 2. Both are efficient trial designs that should expedite further development if evidence of safety and efficacy emerges. Lessons learned from the recruitment challenges in this study will also inform study design of subsequent studies arising from this line of work, and similar studies in RA.

### 3.1 Trial status

Part 1 of the study is completed and part 2 is ongoing using the MTD of seliciclib identified in part 1. Recruitment for part 2 has been slow, necessitating amendments to the background bDMARD (abatacept and tocilizumab also allowed) and DAS28 cut-off inclusion criteria as outlined.

## Acknowledgments

The authors thank all participating sites and investigators for their support with the clinical study and the patients for their participation in the study.

## Author contributions

All authors read and approved the final manuscript.

SS is a principal investigator, contributed to the study design and drafted the manuscript. DDS is a Trial Statistician and drafted the manuscript. JDI is the chief investigator. He conceived the study and designed the trial. MM is Trial Manager and contributed to writing the study protocol. AC was Trial Manager and contributed to study design, protocol development and reviewed the manuscript. SF liaised with the principal investigators to provide information on seliciclib and advise on the appropriate timing for PD sampling. She also assisted with the grant application and conducted the pharmacodynamic analysis of patient samples. MC is the Trial Statistician. He contributed to the design of part 1 and has contributed to the writing of this manuscript. AGP, CDB, WFN, AF and IBM are principal investigators, contributed to the study design and have reviewed the manuscript.
